# Rapid Reverse Purification DNA Extraction Approaches to Identify Microbial Pathogens in Wastewater

**DOI:** 10.3390/microorganisms11030813

**Published:** 2023-03-22

**Authors:** Sarah Schurig, Rea Kobialka, Andy Wende, Md Anik Ashfaq Khan, Phillip Lübcke, Elias Eger, Katharina Schaufler, Arwid Daugschies, Uwe Truyen, Ahmed Abd El Wahed

**Affiliations:** 1 Institute of Animal Hygiene and Veterinary Public Health, Leipzig University, 04103 Leipzig, Germany; 2 Xpedite Diagnostics GmbH, 80687 Munich, Germany; 3 Institute of Pharmacy, University of Greifswald, 17489 Greifswald, Germany; 4 Institute of Infection Medicine, Christian-Albrecht University Kiel, 24105 Kiel, Germany; 5 University Medical Center Schleswig-Holstein, 24105 Kiel, Germany; 6 Institute of Parasitology, Centre for Infectious Disease, Leipzig University, 04103 Leipzig, Germany

**Keywords:** DNA extraction, reverse purification, wastewater, surveillance

## Abstract

Wastewater monitoring became a promising solution in the early detection of outbreaks. Despite the achievements in the identification of pathogens in wastewater using real-time PCR, there is still a lack of reliable rapid nucleic acid extraction protocols. Therefore, in this study, samples were subjected to alkali, proteinase K and/or bead-beating followed by reverse purification magnetic beads-based separation. Wastewater samples spiked with *S. aureus*, *E. coli* and *C. parvum* were used as examples for Gram-positive and -negative bacteria and protozoa, respectively. All results were compared with a spin column technology as a reference method. Proteinase K with bead beating (vortexing with 0.1 mm glass beads for three minutes) was particularly successful for bacterial DNA extraction (three- to five-fold increase). The most useful extraction protocol for protozoa was pre-treatment with proteinase K (eight-fold increase). The selected methods were sensitive as far as detecting one bacterial cell per reaction for *S. aureus*, ten bacterial cells for *E. coli* and two oocysts for *C. parvum*. The extraction reagents are cold chain independent and no centrifuge or other large laboratory equipment is required to perform DNA extraction. A controlled validation trial is needed to test the effectiveness at field levels.

## 1. Introduction

Classical surveillance methods for the identification of endemic or emerging pathogens have a limited significance with increasing populations [1]. Therefore, the monitoring of wastewater became an important tool to tackle the spread of antimicrobial-resistant bacteria [2], poliovirus [3] and SARS-CoV-2 [4], mostly originating from humans and animals. The World Health Organization declared that especially zoonotic microorganisms entering the water cycle from animal excreta are an underestimated hazard for humans and animals as well as the environment [5]. Animal waste represents 80% of the global fecal biomass and is expected to increase in the coming years [6]. This huge amount has an influence on the composition of the microbial profile of surface water, leading to an increased risk of waterborne zoonotic diseases. The exact burden of the environment as a reservoir is difficult to determine due to a lack of data and legislation. Wastewater screening has increased the potential for pathogen surveillance to prospectively reduce the risk of (re)infection of humans and animals, including the Gram-positive bacterium *Staphylococcus aureus* (*S. aureus*), the Gram-negative bacterium *Escherichia coli* (*E. coli)* and the protozoan *Cryptosporidium parvum* (*C. parvum*) [5,7,8,9]. *S. aureus* and *E. coli* are, in many cases, commensal but can cause diseases in both humans and animals. Symptoms of *S. aureus* infections in humans and animals include skin lesions, endocarditis, pneumonia and/or septicemia, in addition to mastitis in animals [10,11]. Depending on the *E. coli* subtype, gastrointestinal, urinary tract infections, meningitis and/or septicemia were recorded [12]. *C. parvum* induces gastrointestinal symptoms, such as diarrhea and/or vomiting, mainly in humans and cattle [13,14].

With water scarcity expected to affect half of the world’s population by 2025, the use of inadequately treated wastewater, e.g., in agriculture and the community, will be a serious and increasing risk to livestock, wildlife and humans [15]. Routine governmental monitoring for contaminants in water does exist in Europe and North America to prevent waterborne diseases. However, they often cover only a small number of pathogens and are limited to culture-based methods [16,17,18]. Nevertheless, culturing pathogens is time-consuming, and, in addition, not all pathogens are culturable. In contrast, molecular diagnostics such as polymerase chain reaction (PCR) are faster and cover wide ranges of microorganisms with a higher specificity and sensitivity [19,20]. For molecular detection, a suitable DNA extraction method is crucial and can influence the outcomes of the PCR assay [21]. Many extraction methods have been established to assure DNA quality. Most of them are costly, time-consuming and require complex electrical equipment [22]. Moreover, introducing variations in nucleic acid extraction protocols can be deemed necessary due to differences in characteristic cellular features among pathogens. Therefore, rapid and simple extraction procedures with minimum handling will lead to better quality nucleic acids, which improves the final molecular results.

The aim of this study was to evaluate a rapid, portable and reliable extraction method that achieves a high amount of extracted DNA for the subsequent detection of pathogens by real-time PCR. Cell suspensions with *S. aureus*, *E. coli* and *C. parvum* were used as representatives for Gram-positive and -negative bacteria and parasites, respectively.

The extraction and purification of DNA that will be applied here relies on the reverse purification principle utilized by the SwiftX DNA kit. Reverse purification means that cell debris and impurities are bound by magnetic beads during the cell lysis step. The debris- and inhibitor-loaded magnetic beads are then attracted by a magnet, which leads to a clearance of the crude lysate. After magnetic separation, the nucleic acids in the supernatant can be used directly for amplification or for storage.

Different pre-treatment options, such as the addition of proteinase K (PK), alkaline treatment (AT) or bead beating (BB) were evaluated separately or in combination. As a reference, a spin column technique was applied in this study.

## 2. Materials and Methods

### 2.1. Sample Preparation

Cell suspensions of *Staphylococcus aureus* subsp. *aureus* (DSM 799) and *Escherichia coli* (DSM 682) were obtained from the German Collection of Microorganisms and Cell Cultures in Braunschweig, Germany, and stored at −80 °C. Before use, these samples were cultivated overnight on Columbia Blood Agar with sheep blood (Thermo Fisher Scientific, Waltham, MA, USA) at 37 °C. Bacterial colonies were removed from the agar plate, resuspended in 700 µL phosphate-buffered saline (PBS) and pelleted (6000× *g*, 3 min) using a microcentrifuge (Eppendorf, Hamburg, Germany). Oocysts of *Cryptosporidium parvum* (provided by the Institute of Parasitology, Centre for Infectious Disease, Leipzig University, Leipzig, Germany) were stored in a solution containing 2% of 250 µg/mL Amphotericin B (Biochrom GmbH, Cambridge, UK), 2% of 10,000 U/mL Penicillin-Streptomycin (Thermo Fisher Scientific, Waltham, MA, USA), 1% of 1 mg/mL Gentamycin (Biochrom GmbH, Cambridge, UK) and 95% PBS at 4 °C. The oocyst solution was also pelleted (16,600× *g*, 5 min). The pellets of *S. aureus*, *E. coli* and *C. parvum* were washed in 200 µL PBS and pelleted at 6000× *g* for three minutes and the supernatant was discarded. *S. aureus*, *E. coli* and *C. parvum* were pelleted separately or combined based on the experiment layout.

### 2.2. DNA Extraction

#### 2.2.1. Spin Column-Based Method

A DNeasy Blood and Tissue Kit (Qiagen, Hilden, Germany) was used as the spin column reference method. DNA of *S. aureus* and *E. coli* was extracted following the manufacturer’s instructions for Gram-positive and -negative bacteria, respectively. The only modification for *S. aureus* was the inclusion of bead beating as the pellet was resuspended in 400 µL PBS and transferred into a Soil Grinding SK38 Precellys Lysing Tube (Bertin, Montigny-le-Bretonneux, France). The suspension was mixed in Precellys 24 Tissue Homogenizer (Bertin, Montigny-le-Bretonneux, France) at 6500 rpm for one minute. A total of 200 µL of the cell suspension was treated as described in the manufacturer’s basic protocol. For *C. parvum*, 100 µL of 1.2% taurocholic acid sodium salt hydrate (Sigma Aldrich, St. Louis, MO, USA) was added to the cell suspension followed by incubation at 37 °C for 120 min. The suspension was pelleted (13,000× *g*, 5 min) and resuspended in 80 µL PBS, 100 µL ATL Buffer (Qiagen, Hilden, Germany) and 20 µL PK. The procedure was continued as the recommendation by the manufacturer’s protocol for Gram-negative bacteria. This was considered a “spin column” reference method.

#### 2.2.2. Rapid Principle of DNA Extraction and Pre-Treatment Options

As the main procedure of DNA extraction, the “SwiftX DNA” Kit (Xpedite Diagnostics, Munich, Germany) was used according to the instructions of the manufacturer. Different pre-treatments were applied as follows: None (I), addition of PK (II), AT at room temperature (III) or 95 °C (IV) and BB (V). No pre-treatment included an incubation step at 95 °C for 15 min, as required by the manufacturer’s basic protocol. In the case of AT, 200 mM sodium hydroxide was added to the lysis mixture, incubated for 15 min and neutralized with 200 mM hydrochloric acid. An overview of the various procedures can be seen in Table 1. The detailed standard operation procedures (SOP) of the DNA extractions can be found in the Supplementary Materials (S1). After the selection of the best pre-treatment procedure, a combination of multiple pre-treatments was tested as follows: PK was combined with AT at 95 °C (protocols II + IV) or BB (protocols II + V). In each run, one sample with no extraction procedure, one sample using only BB and one sample extracted by a spin column reference method with and without BB were included as control.

#### 2.2.3. Bead Beating

For the bead beating tests, DNA of *E. coli* and *S. aureus* was extracted with a combination of BB and PK (II + V). The pellets were resuspended in 300 µL DLN Buffer (Xpedite Diagnostics, Munich, Germany) and transferred into a 2 mL microcentrifuge tube containing 250 mg of glass beads (Sigmund Lindner, Warmensteinbach, Germany). Glass bead sizes were Ø 0.1 mm (0.09–0.15 mm), Ø 0.5 mm (0.4–0.6 mm) or Ø 1.0 mm (0.70–1.30 mm). The suspension was mixed at 2000 rpm in the Vortexer (Heathrow Scientific, Vernon Hills, IL, USA) for three minutes or in the Hulamixer (Thermo Fisher Scientific, Waltham, MA, USA) for five minutes. A total of 200 µL of each cell suspension was transferred into a new microcentrifuge tube, mixed with 10 µL PK and treated using protocol II. In the case of *C. parvum*, DNA was extracted either with protocol II or using PK in combination with 0.1 mm or 0.5 mm glass beads in the Vortexer as described before. The performance of BB for *C. parvum* was also evaluated with prefilled tubes containing 0.1 mm zirconium beads (Benchmark Scientific, Sayreville, NJ, USA).

#### 2.2.4. Limit of Detection (LOD)

**Figure 1:**
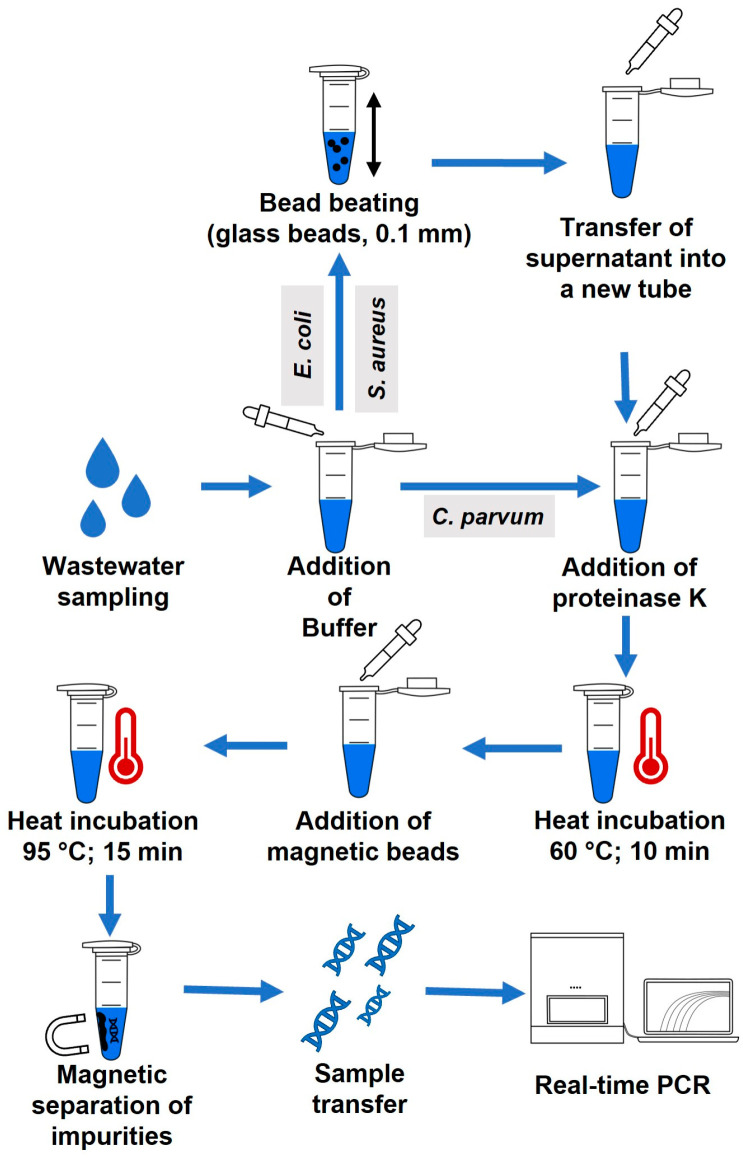
Flowchart of the rapid extraction method selected for determining the limit of detection for *S. aureus*, *E. coli* and *C. parvum*.

A total of 10^7^ (*S. aureus*) and 10^8^ (*E. coli*) bacterial cells and 10^5^ oocysts of *C. parvum* were counted with an Olympus BX40 microscope (Microscopy Technologies, Tokyo, Japan), using a Neubauer Chamber—An amount of 200 µL of wastewater, provided as unfiltered influent water retrieved from the sewage treatment plant Greifswald-Ladebow, Greifswald, Germany, was spiked with the bacterial cells and oocysts. A tenfold serial dilution of the spiked wastewater was prepared. For the DNA extraction of *S. aureus* and *E. coli*, 200 µL of each dilution was mixed with 200 µL DLN Buffer and the procedure was continued as described in Section 2.2.3. using 0.1 mm glass beads and the Vortexer for BB. In the case of *C. parvum*, protocol II (Table 1) with DLN buffer was used. Nucleic acids of each dilution after extraction were tested using real-time PCR. For the determination of LOD, this was repeated three times. The detailed protocols of the DNA extractions can be found in the supplementary (S2). A layout of the final extraction procedure is illustrated in Figure 1.

### 2.3. Real-Time PCR

All extracted samples were screened in real-time PCR operated in a Stratagene Mx3000P Multiplex Quantitative PCR System (Agilent, Santa Clara, CA, USA) according to the pipetting scheme and thermal profile stated in Table 2. The selection of primers and probes, volumes and thermal profiles was based on the study of Luciani et al. for *S. aureus* [23] and Tillman et al. for *E. coli* [24]. In the case of *C. parvum*, the real-time PCR protocol described by Dresley et al. [25] was employed.

### 2.4. Determination of Nucleic Acid Concentration

Nucleic acid concentration was measured using a Nanodrop 2000c (Thermo Fisher Scientific, Waltham, MA, USA) and a Qubit 2.0 Fluorometer in combination with a Qubit dsDNA BR Assay Kit (Thermo Fisher Scientific, Waltham, MA, USA) according to the instructions of the manufacturers.

### 2.5. Statistical Analysis

The LOD with 95% probability and the probability of detection (POD) were determined by the method of Wilrich and Wilrich recommended in the ISO standard 16140-2:2016 using the PODLOD program version 11 (Freie Universität Berlin, Berlin, Germany) [26]. The correlation between nucleic acid measurement by Qubit, Nanodrop and real-time PCR was determined according to the distribution of data using GraphPad Prism version 9.0 (GraphPad Software Inc., San Diego, CA, USA).

Based on the quantification cycle (*C_q_*), the total amount of extracted DNA was calculated using the following formula making the highest *C_q_* adapted to the reagent volume 100%:

$${DNA}_{Norm.ex.}=\frac{1}{200*2^{\left(C_{q1}-{Cq}_{low}\right)}}*V\;{DNA}_{Norm.ex.100\%}=\frac{100*{DNA}_{Norm.ex.}}{{DNA}_{Norm.ex.high}}$$

*DNA_Norm.ex._* = total amount of recovered DNA (%)
*C_q_*_1_ = measured quantification cycle for the sample of interest
*C_q__low_* = lowest quantification cycle of each run
*V* = total volume (µL)
*DNA_Norm.ex._*_100%_ = *DNA_Norm.ex._* normalized to a maximum amount of 100%
*DNA_Norm.ex.high_* = highest amount of *DNA_Norm.ex._* of each run (%)

## 3. Results

### 3.1. Comparison of Various Extraction Procedures

**Figure 2:**
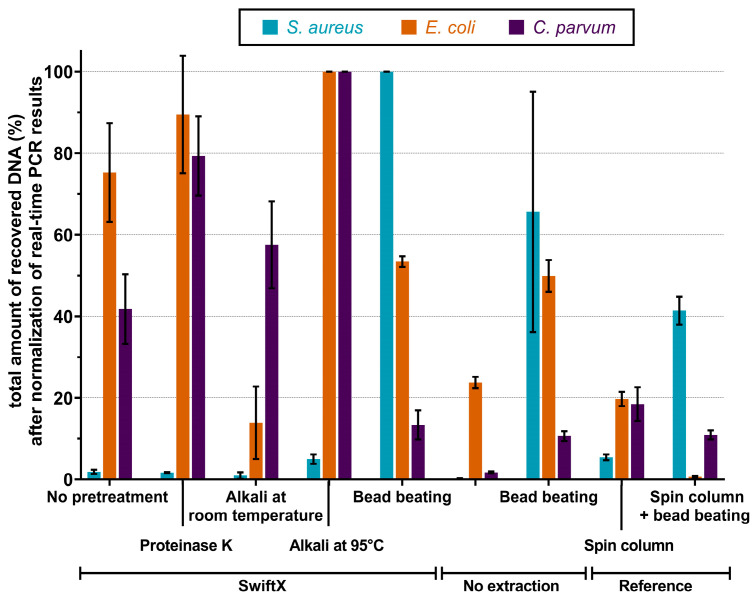
Performance of various extraction protocols with and without pre-treatment. The percentage of recovered DNA for different pre-treatment protocols was measured after the normalization of real-time PCR results. Cell suspension of 10^7^ bacterial cells of *S. aureus*, 10^8^ bacterial cells of *E. coli* or 10^6^ oocysts of *C. parvum* was pelleted and extracted. DNA was measured by real-time PCR. The highest percentage of recovered DNA was achieved using SwiftX with bead beating in the case of *S. aureus*. SwiftX with proteinase K or alkaline treatment for *E. coli* and *C. parvum* revealed the best DNA recovery rate.

Several pre-treatment options were tested to identify the most efficient DNA extraction procedure for *S. aureus*, *E. coli* and *C. parvum*. All tested protocols are summarized in Table 1. For *S. aureus*, the addition of a BB step to the SwiftX protocol resulted in the highest DNA recovery and was more than two-fold higher than the spin column reference method (Figure 2). The combination of PK or AT with the SwiftX protocol resulted in no significant effect on the DNA yield. The most efficient extraction methods for *E. coli* were SwiftX combined with AT at 95 °C or the addition of PK. Surprisingly, DNA recovery rates using the spin column reference method were five times lower (Figure 2). For the extraction of *C. parvum*, the SwiftX protocols showed a zero- to five-fold better performance than the spin column reference protocol. The AT at 95 °C or preincubation with PK increased the amount of recovered DNA compared to the spin column reference method four- and five-fold, respectively (Figure 2).

### 3.2. Evaluation of Combined Pre-Treatment Protocols

**Figure 3:**
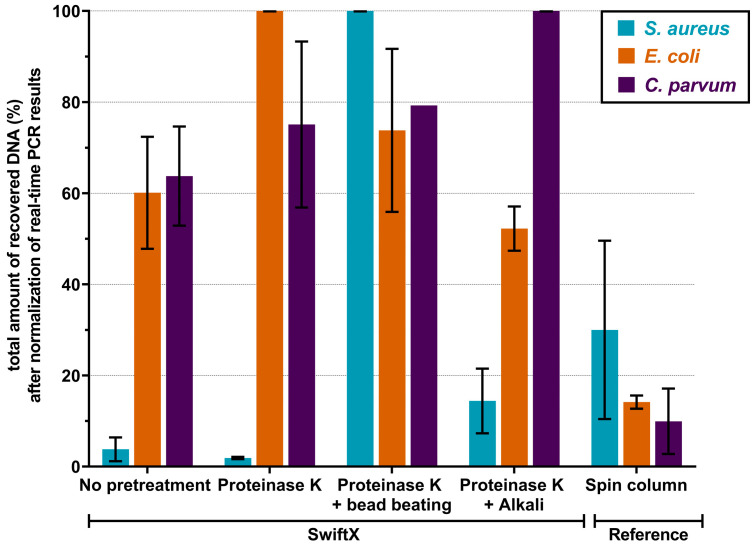
Results of the combination of various pre-treatment protocols. Between 10^6^ and 10^8^ of bacterial cells of either *S. aureus* or *E. coli*, as well as oocysts of *C. parvum*, were prepared to test a maximum dual pre-treatment based on the best performer in Figure 2. Highest percentage of recovered DNA was achieved using SwiftX combined with proteinase K and bead beating for *S. aureus*. For *E. coli*, proteinase K or a combination of proteinase K and bead beating proved to be most efficient, while proteinase K or a combination of proteinase K and either bead beating or alkaline treatment worked the best for *C. parvum*.

The best single pre-treatment protocols (Figure 2) were combined to determine the most effective mixture with SwiftX. Pre-treatments with none, PK, PK + BB and PK + AT at 95 °C were tested. The results are shown in Figure 3. For *S. aureus*, the highest amount of DNA was obtained in the protocol of SwiftX with PK + BB, which was the only method to outperform the spin column reference technique (an increase of DNA amount by a factor of three). In contrast, all combinations of pre-treatment and SwiftX for the extraction of *E. coli* performed three to seven times higher than the spin column reference method. Prominently, a seven- and five-fold increase was achieved for the SwiftX protocol with PK or a combination of PK and BB, respectively. Likewise, the DNA of *C. parvum* was recovered in a six- to ten-fold higher amount with all variations of the SwiftX protocol compared to the spin column reference method. Nevertheless, the best results were achieved using pre-treatment with PK, PK + BB or PK + AT.

### 3.3. Influence of Different Bead-Beating Methods and Glass Bead Sizes on the DNA Extraction Performance

**Figure 4:**
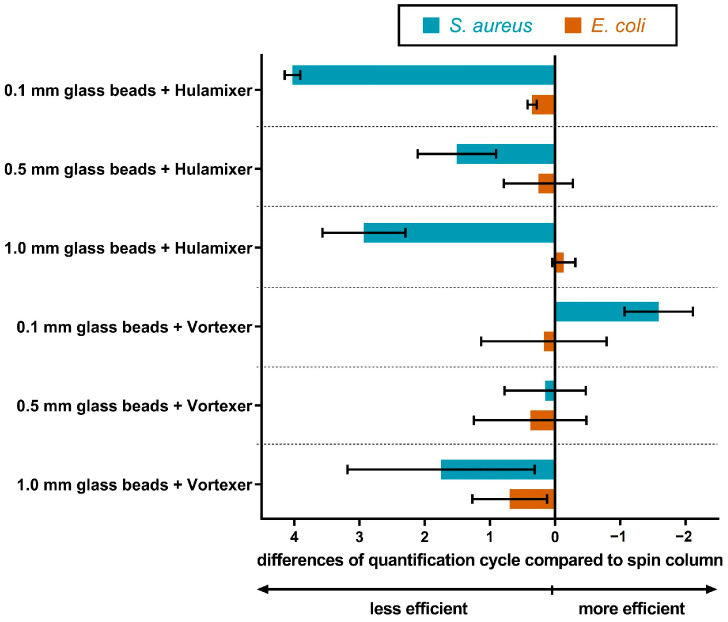
Effect of glass bead size and bead beating force on the extraction performance. To identify the optimal protocol for DNA recovery, SwiftX was combined with bead beating using glass beads with different sizes (0.1 mm, 0.5 mm or 1.0 mm). The real-time PCR results of SwiftX protocols and the spin column reference technique were compared. The use of 0.1 mm glass beads combined with the Vortexer showed the best results for *S. aureus*, while equal performance was achieved for *E. coli*.

DNA was extracted from a bacterial cell suspension of *S. aureus* and *E. coli* using the SwiftX protocol combined with a BB step with glass beads in sizes 0.1 mm, 0.5 mm or 1.0 mm. Additionally, performances of a simple hulamixer or Vortexer for the BB step were compared to each other to avoid the use of an expensive complex BB device. The use of 0.1 mm glass beads together with the Vortexer outperformed all tested extraction methods for *S. aureus* (Figure 4). In contrast, the different BB variations for the DNA extraction of *E. coli* did not show a significant difference. In the case of *C. parvum*, glass beads in sizes 0.1 mm and 0.5 mm, as well as 0.1 mm zirconium beads, were used for DNA extraction with the SwiftX protocol. Results were comparable to the use of SwiftX with PK; no advantages were recorded for *C. parvum* DNA recovery.

### 3.4. Correspondence of Quantification Cycle Determined by Real-Time PCR and Nucleic Acid Concentration Measured by Nanodrop and Qubit

No substantial correlation could be identified between the nucleic acid concentration measured by Nanodrop or Qubit and the *C_q_* values by real-time PCR for *S. aureus*, *E. coli* and *C. parvum*, as shown in S3.

### 3.5. Time and Pipetting Steps of Various Extraction Protocols

To identify the best protocol for rapid extraction time, working steps, reagents volume and number for electrical devices for each protocol were compared. While SwiftX required three to five working steps in a total of 30 or 35 min, the spin column reference method needed more time (up to 270 min) and seven to nine pipetting steps. A BB step needs larger volumes of reagents (400–480 µL) and a tissue homogenizer or Vortexer. A heat block was crucial for all protocols and a high-speed centrifuge was a must for the spin column reference method, while it was not required for the SwiftX technology (Table 3).

### 3.6. Determination of LOD for the Extraction Method of Choice

**Figure 5:**
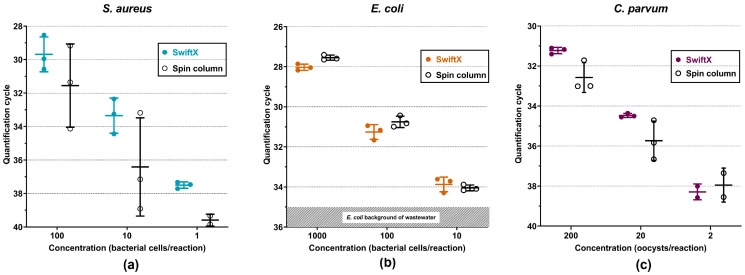
Determination of limit of detection in spiked wastewater samples extracted by the most efficient extraction procedures for *S. aureus* (**a**), *E. coli* (**b**) and *C. parvum* (**c**). SwiftX protocol was combined with proteinase K and a bead-beating step for *S. aureus* and *E. coli*, whereas for *C. parvum*, the proteinase K and SwiftX protocol was applied. Ten-fold serial dilutions of spiked wastewater were screened. After extraction, the quantification cycle of each dilution was measured using real-time PCR. All results were compared to a spin column reference method. Down to one bacterial cell of *S. aureus* per reaction was detected (**a**), while the threshold was ten with *E. coli* (**b**). For *C. parvum*, the limit of detection was two oocysts per reaction (**c**). Because of the *E. coli* pre-existence in wastewater, the shaded zone in (**b**) represents the background values.

**Figure 6:**
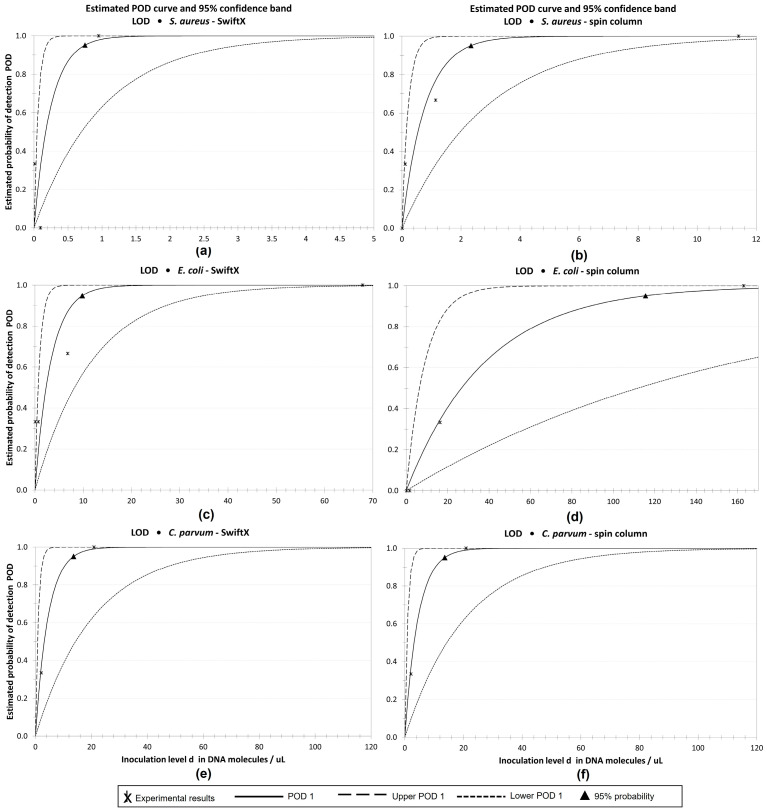
Probability of detection of method of choice for the extraction of *S. aureus* (**a**,**b**), *E. coli* (**c**,**d**) and *C. parvum* (**e**,**f**). Between 10^3^ and 10^6^ bacterial cells or oocysts per microliter were spiked into wastewater in a ten-fold serial dilution. In the case of *S. aureus* and *E. coli*, DNA was extracted using SwiftX in combination with proteinase K and bead beating. For *C. parvum*, the SwiftX protocol was combined in a preincubation with proteinase K. The efficiency of the extraction protocols was compared to a spin column reference method. The probability of the detection function was calculated by the Wilrich and Wilrich model using quantification cycles measured by real-time PCR. The 95% probability is depicted as a triangle. For *S. aureus*, 0.74 copies per reaction with SwiftX (**a**) and 2.33 with the spin column reference method (**b**) were calculated. For *E. coli*, 9.94 and 115.1 copies per reaction were measured for SwiftX (**c**) and the spin column reference method (**d**), respectively. In the case of *C. parvum*, 13.55 copies per reaction were determined for both the SwiftX (**e**) and the spin column reference method (**f**).

The combination of the SwiftX protocol with the use of PK + BB was chosen as the method of choice for *S. aureus* and *E. coli*. For *C. parvum*, the SwiftX protocol including PK treatment was selected. The LOD of the DNA extraction method for each pathogen was determined in a ten-fold serial dilution of spiked wastewater to simulate the real case scenario. An amount between 10^3^ and 10^6^ bacterial cells or oocysts per microliter was used to spike the wastewater and was extracted with the respective protocol using the spin column reference method as control. Down to one bacterial cell of *S. aureus* per reaction was detected by combining the methods of choice with real-time PCR. The detected amount of DNA for each dilution was on average 0.7 log levels higher than the spin column reference method (Figure 5a). The number of detected cells per microliter in each dilution of *E. coli* sample after extraction with SwiftX and PK + BB were comparable to those achieved with the spin column reference method. In lower dilutions, ten DNA molecules per reaction were consistently detected due to the background level of *E. coli* in the wastewater (Figure 5b). Down to two oocysts of *C. parvum* per reaction were detected by both the SwiftX and spin column reference techniques (Figure 5c). With a 95% probability, 0.74 copies per reaction with SwiftX and 2.33 copies per reaction with the spin column reference method were calculated for *S. aureus*. In the case of *E. coli*, 9.94 and 115.1 copies per reaction were identified for SwiftX and the spin column reference method, respectively. For the extraction of *C. parvum* DNA, both methods, the SwiftX protocol and the spin column reference method, achieved 13.55 copies per reaction. The probability of detection curves are shown in Figure 6.

## 4. Discussion

In this study, a portable rapid DNA extraction method based on reverse purification separation was established for the extraction of pathogen DNA from wastewater. The method included a pre-treatment with PK and BB for bacteria and a pre-treatment with PK for protozoa, respectively.

In all experiments, molecular testing (real-time PCR) was selected over culture-based detection methods which required 12 h of incubation and specially trained laboratory staff. Real-time PCR is much faster while enabling a high sensitivity and specificity of the analysis [20,27]. As clearly shown in our study and by other groups, DNA extraction is a very crucial step to obtain a sufficient amount and quality of DNA for real-time PCR, which is instrumental in avoiding false negative results [21,22]. In this regard, it is not only the quality of the assay that affects the sensitivity of the detection method, but also the extraction procedure.

Although all tested combinations of sample pre-treatment led to detectable amounts of DNA, the quality and yield varied among the different treatments. This was expected, as the outer layer structures of the tested pathogens are very different. Gram-positive bacteria such as *S. aureus* have a thick homogeneous cell wall (20–40 nm) containing a wide peptidoglycan layer [28,29]. The cell wall of Gram-negative bacteria such as *E. coli* is much thinner (15 nm) and composed of an inner membrane, a thin peptidoglycan monolayer and an outer membrane [28,29,30]. The oocyst wall of *C. parvum* is very rigid. It is 40 nm thick and contains an inner filamentous and an outer glycoprotein layer [31]. When PK was used separately or in combination, the amount of extracted DNA increased, especially for *E. coli* and *C. parvum*, as the PK eliminates interfering proteins in the solution and protects free DNA by inactivating DNases [32]. Moreover, the protease degrades the filamentous layer of the oocyst wall resulting in an increase in extraction efficiency for *C. parvum* [31]. For *S. aureus*, PK was likely not adequate to disrupt the thick cell wall of the Gram-positive bacterium. An additional BB step proved to be essential for the extraction of DNA from *S. aureus*. These results are in accordance with data obtained from previous studies [33,34,35]. Modification of the BB method improved the extraction efficiency for *E. coli*. This emphasizes that bead size, BB duration and BB device can influence the DNA yield and need to be adjusted to the specific pathogen. Likewise, Proctor et al. have previously made the same observation [36]. With the use of the reverse purification principle, the cleared lysate still contains certain organic and inorganic compounds, which can influence measurements by real-time PCR [37], which is why an applicability testing of reverse purification-based methods is advised before implementation in the subsequent workflow. A previous study by Hansen et al. reported a significant inhibitory effect when using the SwiftX DNA kit for direct DNA extraction from fecal samples [38]. Thus, our observation that there was no difference in the performance of DNA extraction from PBS and wastewater, respectively, was somewhat surprising. This may be explained by the significantly lower concentration of fecal matter in wastewater compared to actual fecal samples. In our case, the lysate after DNA extraction was clear and colorless. However, direct extraction of DNA from solid fecal samples may not be successful as substances such as bilirubin, which gives the brownish fecal coloration and can interfere with the real-time PCR, might only be insufficiently removed [39].

The developed method comprises several advantages. First, DNA can be extracted very rapidly with a maximum working time of 35 min. Second, the procedure involves no more than four steps and is therefore easy to learn and to implement. Third, it requires only two basic electrical devices: a heating block and a small Vortexer. Both can be operated as part of a mobile suitcase lab under field conditions [40]. Fourth, the reagents are heat stable and do not require refrigeration or freezer storage, which again improves field applicability. In contrast, the spin column reference method requires an expensive tissue homogenizer, a high-speed centrifuge and more handling time.

Down to one bacterial cell per reaction was detected for *S. aureus*, ten bacterial cells for *E. coli* and two oocysts for *C. parvum*, which was similar to the spin column reference method. Establishing a highly sensitive extraction and detection procedure is crucial, especially in the case of water testing, where pathogen concentrations might be low [41,42]. Rainfall and runoff, as well as regional and seasonal differences, can cause huge variations in the pathogen’s quantity and may result in a drastic dilution effect of the wastewater [43]. Despite the efficacy of the method established in this study, a concentration step (e.g., ultrafiltration) might be needed in a future application to avoid false negative results. Additionally, this would provide the capability to study larger volumes of wastewater. In the experiments performed to determine the best pre-treatment options, the SwiftX protocols resulted in an up to 10-fold increase of recovered DNA compared to the spin column reference method. The differences can be explained by the loss of DNA due to the number of working steps and the need for a high-speed centrifuge in the reference method. In the case of the reverse purification method, fewer working and sample transfer steps are required, which results in less stress and loss of DNA molecules.

Despite the many advantages of the reverse purification methods, their compatibility with the sample type of interest must be examined to avoid any potential inhibitors impairing the downstream applications. The method was successfully implemented for nucleic acid extraction from skin [44], feces [38], urine [45], cervicovaginal lavage [46] and blood [40], but in combination with isothermal amplification methods, which are known to resist several common inhibitors of real-time PCR chemistries [47]. Sample purity and the concentration of nucleic acids are usually measured by Nanodrop or Qubit. In our experiment, no correlation was found between the results of these measurements and the detectability in the molecular assays. Even though nucleic acid concentrations could be measured by Nanodrop, the dynamic ranges and the ratios of wavelengths indicate that they are due to background noise. Quantification of DNA concentration by Nanodrop is based on UV absorption measurement at a DNA-specific wavelength, while the Qubit instrument conducts a fluorescence-based measurement of a DNA-bound dye. Li and Wu et al. found that the UV absorption method and the fluorescence method can be affected by the presence of organic and chemical contaminants [48]. Since in the reverse purification both remnants of cells and lysis buffer components remain as background, the measurements of DNA concentrations by Nanodrop and Qubit have likely been influenced. Consequently, the use of DNA concentration measurement in combination with the established extraction method is not recommended. Overall, our findings suggest that the rapid extraction methods formulated in this study are field-applicable and need further controlled trials to confirm. Moreover, the effectiveness of the extraction of nucleic acids also needs to be determined in the detection of antimicrobial resistance genes and viruses, as these are important contributors to the health risks imposed by wastewater.

## Figures and Tables

**Table 1:** Overview of working steps for different extraction protocols.

Protocol ID	Extraction Method	Buffer + Magnetic Beads	Proteinase K	Alkali	Bead Beating	10 min 60 °C	15 min RT	15 min 95 °C	Magnetic Separation
I	No pre-treatment	x						x	x
II	Proteinase K	x	x			x		x	x
III	Alkaline treatment RT	x		x			x		x
IV	Alkaline treatment 95 °C	x		x				x	x
V	Bead beating	x			x			x	x

RT = room temperature.

**Table 2:** Real-time PCR protocols for *S. aureus*, *E. coli* and *C. parvum*.

	Mastermix	Thermal Profile ^2^
*S. aureus*	10.00 µL QuantiNova Probe Master Mix	Denaturation: 95 °C; 120 s
	(Qiagen, Hilden, Germany)	Amplification (45 cycles):
	0.64 µL Primer/Probe Mix	Denaturation: 95 °C; 5 s
	1.28 µM forward primer StaphF	Annealing and extension: 60 °C; 30 s
	1.28 µM reverse primer StaphR	
	0.64 µM probe StaphP	
	8.36 µL PCR clean water	
	1.00 µL template	
*E. coli*	10.00 µL QuantiNova Probe Master Mix	Denaturation: 95 °C; 120 s
	(Qiagen, Hilden, Germany)	Amplification (35 cycles) ^1^:
	1.00 µL Primer/Probe Mix	Denaturation: 94 °C; 15 s
	0.40 µM forward primer gadE-F	Annealing and extension: 56 °C; 45 s
	0.40 µM reverse primer gadE-R	
	0.20 µM probe gadE-Probe	
	8.00 µL PCR clean water	
	1.00 µL template	
*C. parvum*	12.50 µL Maxima probe/ROX qPCR Master Mix	Denaturation: 95 °C; 900 s
	(Thermo Fisher Scientific, Waltham, MA, USA)	Amplification (40 cycles):
	0.30 µL forward primer (0.30 µM) CP_hsp70_fwd	Denaturation: 95 °C; 15 s
	0.90 µL forward primer (0.90 µM) CP_hsp70_rvs	Annealing and extension: 60 °C; 60 s
	0.20 µL forward primer (0.20 µM) Hsp70_snd	
	6.10 µL PCR clean water	
	5.00 µL template	

^1^ Forty cycles for the determination of limit of detection; ^2^ Thermal profile of *C. parvum* was used in case of multiplexing with *S. aureus.*

**Table 3:** Number of working steps, time (min.), added volume of reagents (in µL) and the number of additional electric devices for different extraction protocols for *S. aureus*, *E. coli* and *C. parvum*.

Extraction Method	Working Steps	Time (min.)	Added Reagents (µL)	Use of Electric Devices
SwiftX + no pre-treatment	3	30	200	-
SwiftX + proteinase K	3	30	200	-
SwiftX + proteinase K + bead beating	4	35	480	Tissue homogenizer (or Vortexer)
SwiftX + proteinase K + alkali 95 °C	5	35	300	-
Spin column				
* S. aureus*	7	40	400	Centrifuge + tissue homogenizer
* E. coli*	6	150	200	Centrifuge
* C. parvum*	9	270	200	Centrifuge

## Data Availability

All data and standard operation procedures are provided in the manuscript or the Supplementary Materials.

## Supplementary Materials

The following supporting information can be downloaded at: https://www.mdpi.com/article/10.3390/microorganisms11030813/s1, S1: Standard operation protocols for separated pre-treatment options; S2: Best performing extraction protocols; S3: Comparison of quantification cycle and nucleic acid concentration.
