# Supplementary material for: Rapid Reverse Purification DNA Extraction Approaches to Identify Microbial Pathogens in Wastewater
Source: Microorganisms. 2023 Mar 22;11(3):813. doi: 10.3390/microorganisms11030813 (PMC10056086; doi:10.3390/microorganisms11030813)
Supplement: Supplementary file 1 [file microorganisms-11-00813-s001.zip › S1 - Standard operation protocols for separated pre-treatment options.pdf]

S1 Standard operation protocols for separated pre-treatment options.

I SwiftX + no pre-treatment:

1. Resuspend pellet in 170 µL DL buffer (Xpedite Diagnostics GmbH, Germany).
2. Shake or vortex Beads A (Xpedite Diagnostics GmbH, Germany) for 30 seconds to ensure homogeneous suspension. Add 30µL of Beads A to the sample mixture. Mix well by vortexing the tube.
3. Incubate lysis mixture at 95°C for 15 minutes in a HLC Heating-ThermoMixer (DITABIS, Pforzheim, Germany).
4. Remove condensate from the lid before opening by shaking down.
5. Place sample tube into a magnetic stand at room temperature for one minute to let the magnetic particles separate.
6. Open the lid while the tube remains in the magnetic stand and transfer the supernatant into a new tube for storage or use in downstream applications.

II SwiftX + proteinase K:

1. Resuspend pellet in 160 µL DL buffer (Xpedite Diagnostics GmbH, Germany).
2. Add 10 µL proteinase K (Qiagen, Hilden, Germany) and incubate cell suspension at 60°C for 10 minutes in a HLC Heating-ThermoMixer (DITABIS, Pforzheim, Germany).
3. Shake or vortex Beads A (Xpedite Diagnostics GmbH, Germany) for 30 seconds to ensure homogeneous suspension. Add 30µL of Beads A to the sample mixture. Mix well by vortexing the tube.
4. Incubate lysis mixture at 95°C for 15 minutes in a HLC Heating-ThermoMixer (DITABIS, Pforzheim, Germany).
5. Remove condensate from the lid before opening by shaking down.
6. Place sample tube into a magnetic stand at room temperature for one minute to let the magnetic particles separate.
7. Open the lid while the tube remains in the magnetic stand and transfer the supernatant into a new tube for storage or use in downstream applications.

III SwiftX + alkaline treatment at RT:

1. Resuspend pellet in 90 µL DL buffer (Xpedite Diagnostics GmbH, Germany) and 90 µL of 200 mM sodium hydroxide.
2. Incubate lysis mixture at RT for 15 minutes.
3. Shake or vortex Beads A (Xpedite Diagnostics GmbH, Germany) for 30 seconds to ensure homogeneous suspension. Add 30µL of Beads A to the sample mixture. Mix well by vortexing the tube.
4. Add 90 µL of 200 mM hydrochloric acid to the suspension and incubate suspension at RT for three minutes.
5. Remove condensate from the lid before opening by shaking down.
6. Place sample tube into a magnetic stand at room temperature for one minute to let the magnetic particles separate.
7. Open the lid while the tube remains in the magnetic stand and transfer the supernatant into a new tube for storage or use in downstream applications.

IV SwiftX + alkaline treatment at 95 °C:

1. Resuspend pellet in 90 µL DL buffer (Xpedite Diagnostics GmbH, Germany) and 90 µL of 200 mM sodium hydroxide.
2. Incubate lysis mixture at 95 °C for 15 minutes in a HLC Heating-ThermoMixer (DITABIS, Pforzheim, Germany).
3. Shake or vortex Beads A (Xpedite Diagnostics GmbH, Germany) for 30 seconds to ensure homogeneous suspension. Add 30µL of Beads A to the sample mixture. Mix well by vortexing the tube.
4. Add 90 µL of 200 mM hydrochloric acid to the suspension and incubate suspension at RT for three minutes.
5. Remove condensate from the lid before opening by shaking down.
6. Place sample tube into a magnetic stand at room temperature for one minute to let the magnetic particles separate.
7. Open the lid while the tube remains in the magnetic stand and transfer the supernatant into a new tube for storage or use in downstream applications.

V SwiftX + bead beating:

1. Resuspend pellet in 400 µL PBS.
2. Transfer mixture into a Soil Grinding SK38 Precellys Lysing Tube (Bertin, Montigny-le-Bretonneux, France) and mix at 6500 rpm for one minute in the Precellys 24 Tissue Homogenizer (Bertin, Montigny-le-Bretonneux, France).
3. Transfer 200 µL of the suspension in a new microcentrifuge tube and add 160 µL of DL buffer (Xpedite Diagnostics GmbH, Germany).
4. Shake or vortex Beads A (Xpedite Diagnostics GmbH, Germany) for 30 seconds to ensure homogeneous suspension. Add 30µL of Beads A to the sample mixture. Mix well by vortexing the tube.
5. Incubate lysis mixture at 95°C for 15 minutes in a HLC Heating-ThermoMixer (DITABIS, Pforzheim, Germany).
6. Remove condensate from the lid before opening by shaking down.
7. Place sample tube into a magnetic stand at room temperature for 1 minute to let the magnetic particles separate.
8. Open the lid while the tube remains in the magnetic stand and transfer the supernatant into a new tube for storage or use in downstream applications.
