# Supplementary material for: Rapid Reverse Purification DNA Extraction Approaches to Identify Microbial Pathogens in Wastewater
Source: Microorganisms. 2023 Mar 22;11(3):813. doi: 10.3390/microorganisms11030813 (PMC10056086; doi:10.3390/microorganisms11030813)
Supplement: Supplementary file 1 [file microorganisms-11-00813-s001.zip › S2 - Best performing extraction protocols.pdf]

**S2 Best performing extraction protocols.**

**SwiftX + proteinase K:**

1. Add 160 µL DLNbuffer (Xpedite Diagnostics GmbH, Germany) to 200 µl wastewater.
2. Add 10 µL proteinase K (Qiagen, Hilden, Germany) and incubate cell suspension at 60°C for 10 minutes.
3. Shake or vortex Beads A (Xpedite Diagnostics GmbH, Germany) for 30 seconds to ensure homogeneous suspension. Add 30µL of Beads A to the sample mixture. Mix well by vortexing the tube.
4. Incubate lysis mixture at 95°C for 15 minutes.
5. Remove condensate from the lid before opening by shaking down.
6. Place sample tube into a magnetic stand at room temperature for 1 minute to let the magnetic particles separate.
7. Open the lid while the tube remains in the magnetic stand and transfer the supernatant into a new tube for storage or use in downstream applications.

**SwiftX + bead beating:**

1. Add 200 µL DLN buffer (Xpedite Diagnostics GmbH, Germany) to 200 µL wastewater.
2. Transfer lysis mixture into a 2.0 mL microcentrifuge tube with 250 mg glass beads.
3. Bead beating in the Vortexer for 3 minutes at 2000 rpm.
4. Transfer 200 µL of the lysis mixture into a new 1.5 ml microcentrifuge tube.
5. Add 10 µL proteinase K (Qiagen, Hilden, Germany) and incubate cell suspension at 60°C for 10 minutes.
6. Shake or vortex Beads A (Xpedite Diagnostics GmbH, Germany) for 30 seconds to ensure homogeneous suspension. Add 30µL of Beads A to the sample mixture. Mix well by vortexing the tube.
7. Incubate lysis mixture at 95°C for 15 minutes.
8. Remove condensate from the lid before opening by shaking down.
9. Place sample tube into a magnetic stand at room temperature for 1 minute to let the magnetic particles separate.
10. Open the lid while the tube remains in the magnetic stand and transfer the supernatant into a new tube for storage or use in downstream applications.
