# Supplementary material for: Rapid Reverse Purification DNA Extraction Approaches to Identify Microbial Pathogens in Wastewater
Source: Microorganisms. 2023 Mar 22;11(3):813. doi: 10.3390/microorganisms11030813 (PMC10056086; doi:10.3390/microorganisms11030813)
Supplement: Supplementary file 1 [file microorganisms-11-00813-s001.zip › S3 - Comparison of quantification cycle and nucleic acid concentration.pdf]

S3 Comparison of cycle threshold and nucleic acid concentration measured by Nanodrop and Qubit for cell suspension of *S. aureus*, *E. coli* and *C. parvum* after extraction. DNA concentration by Nanodrop and Qubit was not an indication of good performance in real-time PCR.

	S. aureus					***E. coli***					***C. parvum***				
	**Nucleic acid concentration (ng/µl)**		**260/280**	**260/230**	**Quanti-fication cycle**	**Nucleic acid concentration (ng/µl)**		**260/280**	**260/230**	**Quanti-fication cycle**	**Nucleic acid concentration (ng/µl)**		**260/280**	**260/230**	**Quanti-fication cycle**
**Extraction protocol**	**Qubit**	**Nanodrop**				**Qubit**	**Nanodrop**				**Qubit**	**Nanodrop**			
**No pre-treatment**	15.55 (± 1.20)	59.10 (± 83.58)	0.61 (± 0.06)	0.18 (± 0.04)	20.92 (± 1.07)	3.68 (± 0.57)	72.80 (± 35.07)	0.62 (± 0.01)	0.20 (± 0.01)	15.52 (± 0.58)	13.40 (± 0.50)	0.00 (± 0.92)	0.02 (± 0.13)	- 0.03 (± 0.09)	27.23 (± 0.30)
**Proteinase K**	35.50 (± 0.71)	323.00 (± 216,09)	0.75 (± 0.04)	0.18 (± 0.01)	21.07 (± 0.53)	3.15 (± 0.77)	53.10 (± 27.29)	0.70 (± 1.17)	0.27 (± 0.09)	15.27 (± 0.11)	13.80 (± 0.10)	0.00 (± 6.15)	0.03 (± 0.34)	- 1.11 (± 1.86)	26.30 (± 0.42)
**Alkaline treatment RT**	20.65 (± 2.76)	25.10 (± 8.49)	0.61 (± 0.03)	0.12 (± 0.01)	22.79 (± 2.09)	0.76 (± 1.07)	31.15 (± 6.01)	0.58 (± 0.01)	0.14 (± 0.01)	18.70 (± 1.34)	12.80 (± 0.40)	0.00	0.42	0.45 (± 0.16)	27.35 (± 0.33)
**Alkaline treatment 95°C**	24.45 (± 0.21)	41.60 (± 36.63)	1.21 (± 0.58)	0.50 (± 0.46)	20.07 (± 0.32)	1.81 (± 0.57)	24.05 (± 0.78)	0.63 (± 0.02)	0.16 (± 0.01)	15.69 (± 0.35)	13.30 (± 0.80)	0.00	0.41 (± 0.01)	0.31 (± 0.10)	26.54 (± 0.59)
**Bead beating**	15.55 (± 0.35)	23.75 (± 4.74)	0.84 (± 0.27)	0.18 (± 0.06)	17.13 (± 0.66)	1.33 (± 0.33)	18.25 (± 13.36)	0.84 (± 0.46)	0.12 (± 0.01)	17.59 (± 0.31)	14.70 (± 0.40)	0.00	0.39 (± 0.02)	- 0.53 (± 1.98)	30.89 (± 0.21)
**No extraction**	13.80 (± 0.28)	7.10 (± 1.27)	2.69 (± 0.82)	- 0.76 (± 0.32)	23.88 (± 0.42)	15.60 (± 4.95)	56.15 (± 79.41)	0.57 (± 0.02)	0.18 (± 0.11)	17.18 (± 0.43)	13.00 (± 0.20)	0.00	0.85 (± 0.11)	0.36 (± 0.03)	31.83 (± 0.79)
**Only bead beating**	14.50 (± 1.98)	16.40 (± 14.14)	2.14 (± 0.11)	1.75 (± 0.63)	16.81 (± 0.01)	1.74 (± 0.05)	23.95 (± 33.87)	1.72 (± 0.04)	- 2.71 (± 3.42)	17.11 (± 0.23)	13.00 (± 0.80)	4.60 (± 1.27)	1.21 (± 0.07)	0.49 (± 0.02)	30.20 (± 0.76)
**Spin column**	16.80 (± 2.40)	0.00	0.18 (± 0.04)	0.63 (± 0.03)	19.34 (± 0.47)	5.38 (±1.17)	1.70 (± 1.98)	0.73 (± 1.54)	- 0.05 (± 0.06)	17.45 (± 0.47)	13.50 (± 0.10)	0.00	0.54 (± 0.08)	0.38 (± 0.58)	28.41 (± 0.92)
**Spin column + bead beating**	15.35 (± 0.49)	9.20 (± 4.10)	0.40 (± 10.51)	- 6.87 (± 8.51)	17.40 (± 0.54)	0.00	0.00	1.02 (± 0.21)	0.09 (± 0.01)	23.34 (± 0.11)	13.20 (± 0.60)	0.00	0.70 (± 0.11)	0.42 (± 0.11)	30.16 (± 0.74)
**Pairwise correlation of nucleic acid concentration with corresponding C_q[1]**	0.228	0.087				- 0.548	- 0.468				- 0.09	0.493			

[1] in term of correlation coefficient r

RT = room temperature

C_q = quantification cycle
